# The role of dairy consumption in the relationship between wealth and early life physical growth in India: evidence from multiple national surveys

**DOI:** 10.1186/s12889-023-17520-8

**Published:** 2024-01-05

**Authors:** Franciosalgeo George, L Naga Rajeev, Sulagna Bandyopadhyay, Jeswin Baby, Srishti Sinha, Harshpal Singh Sachdev, Anura V Kurpad, Tinku Thomas

**Affiliations:** 1 Division of Epidemiology, Biostatistics, and Population Health, St. John’s Research Institute, St. John’s National Academy of Health Sciences, Bangalore, India; 2 Department of Pediatrics and Clinical Epidemiology, Sitaram Bhartia Institute of Science and Research, New Delhi, India; 3 Division of Nutrition, St. John’s Research Institute, St. John’s National Academy of Health Sciences, Bengaluru, India; 4 Department of Statistical Sciences, Kannur University, Kerala, India; 5 Department of Physiology, St John’s Medical College, Bengaluru, India; 6 Department of Biostatistics, St John’s Medical College, Bengaluru, India; 7 Research and Development Cell, Caritas Hospital and Institute of Health Sciences, Kottayam, India

**Keywords:** Child nutrition, 6–59 months old children, Height-for-age z-scores, Dairy consumption, Statistical matching, Path models.

## Abstract

**Introduction:**

Prevalence of undernutrition continues to be high in India and low household wealth is consistently associated with undernutrition. This association could be modified through improved dietary intake, including dairy consumption in young children. The beneficial effect of dairy on child growth has not been explored at a national level in India. The present analyses aimed to evaluate the direct and indirect (modifying association of household level per adult female equivalent milk and milk product consumption) associations between household wealth index on height for age (HAZ) and weight for age (WAZ) in 6-59 months old Indian children using data from of nationally representative surveys.

**Methods:**

Two triangulated datasets of two rounds of National Family Health Survey, (NFHS-3 and 4) and food expenditure (National Sample Survey, NSS61 and 68) surveys, were produced by statistical matching of households using Non-Iterative Bayesian Approach to Statistical Matching technique. A Directed Acyclic Graph was constructed to map the pathways in the relationship of household wealth with HAZ and WAZ based on literature. The direct association of wealth index and its indirect association through per adult female equivalent dairy consumption on HAZ and WAZ were estimated using separate path models for each round of the surveys.

**Results:**

Wealth index was directly associated with HAZ and WAZ in both the rounds, but the association decreased from NFHS-3 (β_HAZ_: 0.145; 95% CI: 0.129, 0.16) to NFHS-4 (β_HAZ_: 0.102; 95%CI: 0.093, 0.11). Adult female equivalent milk intake (increase of 10gm/day) was associated with higher HAZ (β_NFHS-3=0.001;95% CI: 0, 0.002; β_NFHS-4=0.002;95% CI: 0.002, 0.003) but had no association with WAZ. The indirect association of wealth with HAZ through dairy consumption was 2-fold higher in NFHS-4 compared to NFHS-3.

**Conclusions:**

The analysis of triangulated survey data shows that household level per- adult female equivalent dairy consumption positively modified the association between wealth index and HAZ, suggesting that regular inclusion of milk and milk products in the diets of children from households across all wealth quintiles could improve linear growth in this population.

**Supplementary Information:**

The online version contains supplementary material available at 10.1186/s12889-023-17520-8.

## Introduction

Despite varying levels of economic development, undernutrition continues to be a public health problem in India, where 36% and 32% of children under 5 y are reported to be stunted and underweight, respectively, based on the latest nationally representative National Family Health Survey (NFHS-5, 2019-21) [1]. Stunting and other forms of undernutrition are associated with morbidity, suboptimal physical growth and cognitive impairment, and increased risk of developing chronic diseases in early adulthood [2]. Among the multifactorial drivers, poor household wealth status has been consistently identified as a risk factor for stunting [3, 4]. However, it could actually be a proxy or a precursor to the true exposures of stunting such as poor dietary intakes [5–7], poorer access to health etc. and inter-generational effects of low wealth such as low maternal height [8–10], and maternal education [5, 11]. In an analyses of national Indian survey data (NFHS-4 2014-15), household wealth-index was strongly associated with undernutrition such that the prevalence of stunting and underweight were higher in households in the lower wealth quintiles compared to the upper quintiles [5] and this expands to a geographical scale where the prevalence is 20% higher in the least developed districts (~ 45%) compared to the most developed (~ 27%) districts of India [12].

Dietary nutrient inadequacy has been identified as an immediate underlying risk factor for childhood undernutrition [13]. Several observational and intervention trials have shown that inclusion of animal source foods (ASFs) on regular basis is beneficial for linear growth [14–17]. This is possibly due to the relatively higher density of energy, protein, fat and micronutrients in ASFs, the positive regulators of growth outcomes [16, 18]. There is a dearth of national level dietary intake data in India, including milk consumption, that can be utilized to examine this association at the population level. Instead, diet diversity score is often used to examine the association of poor dietary intake and nutritional status.

A diverse diet is expected to ensure adequate intake of essential nutrients for growth and development. However, diet diversity scores which are used in the large surveys may not be effective in examining the association of diet and nutritional status in children < 2y, as there may be no association between diet diversity and nutritional status [19]. Moreover, household wealth dictates these associations as it is a determinant of dietary intake in India [3–5].

In the absence of a single nationally representative survey that simultaneously collected data on food and nutrient intakes using precise methods and anthropometric measurements in Indian children, the present analyses aimed to evaluate the direct association between household wealth index as a composite measure of living standards on nutritional status such as height for age (HAZ) and weight for age (WAZ) in children aged 6–59 months and the association of wealth that is explained by household level per adult female equivalent(AFE) milk and milk products consumption on these associations in a triangulated dataset of two different Indian national surveys (NFHS and NSS survey) (NFHS-4 2015-16 with NSS-68 2011-12, and NFHS-3 2004-05 with NSS-61 2005-06) using a robust framework of possible causal associations between wealth and undernutrition identified from the available literature. The temporal changes in the direct and indirect association of wealth on nutritional status were examined using two rounds of diet and health surveys triangulated data.

## Methods

The association of milk intake with nutritional status of children was examined by creating a synthetic data set of milk intake and nutritional status in children aged 6–59 months by statistically matching households in the National Family Health Survey 4 (NFHS-4) and National Sample Survey 68 (NSS-68) as well as National Family Health Survey 3 (NFHS-3) and National Sample Survey 61 (NSS-61).

### Data sources

The individual level data on height and weight measurements of children aged 6–59 months were obtained from NFHS-3 [20] and NFHS-4 [21] along with household sociodemographic and maternal characteristics. The milk intake data were obtained from the National Sample Survey (NSS), rounds 61 and 68 [22, 23].

The NFHS-4 was conducted between January 2015 and December 2016, covering both urban and rural areas across 29 states and 7 union territories of India [21]. Similarly, NFHS-3 was conducted between November 2005 to August 2006, covering urban and rural regions in 29 Indian states, but not the union territories [20]. The anthropometric measurements in NFHS-4 were obtained from a total of 206 073 children aged 6–59 months, from 156 038 households. In NFHS-3, data were available for 37,960 children from 28,497 households. The sample size in NFHS-4 was much larger than in NFHS-3 due to the design differences between the two surveys. NFHS-3 was designed for state-level estimates of various health indicators, while NFHS-4 aimed to provide district-level (a sub-division of state) estimates, requiring the larger sample size. Maternal height and height of children aged 24–59 months were measured using Seca 213 stadiometer. The recumbent length of children < 2y or less than 85 cm was measured using the Seca 417 infantometer [20, 21]. Weight was measured on an electronic SECA 874 flat scale designed for mobile use. The mother or caretaker was weighed first when weighing very young children. While holding the kid, the mother or caretaker was weighed again. The mother’s stored weight was deducted, and the baby’s weight was displayed on the scale, with the help of an automatic two-in-one adjustment button [20, 21]. Stunting was defined as a child’s height-for-age z-score (HAZ) less than − 2 and underweight defined as child’s weight-for-age z-score (WAZ) less than − 2 computed using World Health Organization (WHO) child growth reference standard [24]. Children with extreme HAZ (<-6 or > 6) or extreme WAZ (<-6 or > 5) or with missing values for either HAZ or WAZ were eliminated from the analysis [24]. In NFHS-3, a total of 5,459 children were excluded due to missing values for HAZ or WAZ, while in NFHS-4, 15,785 children were excluded for the same reason. Wealth scores based on the household possession of consumer goods were derived using principal component analysis. National level wealth quintiles are formed by dividing the population into five equal categories based on their wealth scores. Wealth indices were those available in the NFHS data sets and were not computed for the purpose of this study. Details of the computation of wealth quintiles are available at demographic and health surveys website [25]. After excluding missing values and outliers from all variables under consideration, we used data on 24,670 and 132,767 children, respectively from NFHS- 3 and NFHS-4 for the analysis. The details of exclusion of records from analysis are provided in supplementary material (Supplementary file 1 Fig. 1(a) and 1(b)). A comparison between the demographic characteristics of the excluded and included data, for age, sex, wealth, and place of residence showed that all variables except mean age of child were comparable between the excluded and included data sets in both NFHS-3 (Supplementary file 1 Table 1) and NFHS-4 (Supplementary file 1 Table 2). Mothers of children under 2 years were asked to report the foods belonging to seven different food groups that were fed to their children. A list of 21 foods were presented to the mothers. The food groups were “grains, roots, and tubers”, “legumes and nuts”, “dairy products”, “flesh foods”, “eggs”, “vitamin A rich fruits and vegetables”, “other fruits and vegetables”. The diet diversity score for each child was the number of food groups consumed (if any one food in a food group was consumed in any quantity, then the food group was marked as consumed) in the last 24 h and the score ranged from zero to seven.

**Fig. 1:**
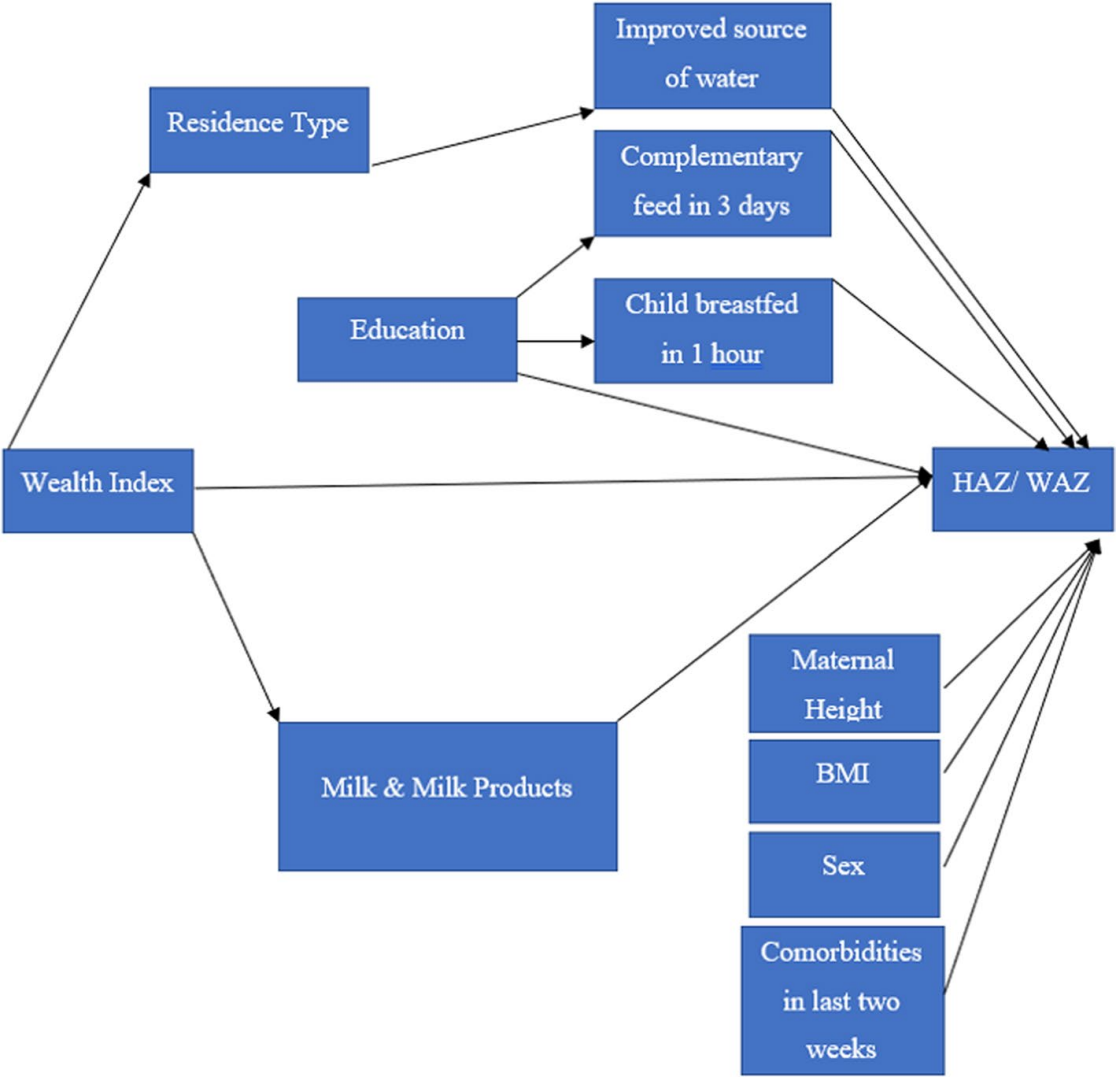
Conceptual framework representing a direct association between the wealth and health outcomes and this association explained through other variables

**Table 1:** Sociodemographic characteristics of the NFHS-3 (2004–2005) and NFHS-4 (2014–2015) samples

	NFHS-3	NFHS-4
Characteristics	*N* = 24,670^1^	*N* = 132,767^1^
Age in Years		
< 1 Year	3,810 (15%)	20,108 (15%)
1–3 Year	12,899 (52%)	67,819 (51%)
3–5 Year	7,961 (32%)	44,840 (34%)
Mother’s BMI		
< 18.5	9,743 (39%)	32,912 (25%)
18.5–24.9	12,935 (52%)	77,664 (58%)
>=25	1,992 (8.1%)	22,191 (17%)
Residence Type		
Urban	6,513 (26%)	39,171 (30%)
Rural	18,157 (74%)	93,596 (70%)
Mother’s Education		
No Education	11,628 (47%)	37,131 (28%)
Primary	3,487 (14%)	18,052 (14%)
Secondary	8,106 (33%)	62,414 (47%)
Higher	1,449 (5.9%)	15,170 (11%)
Wealth Index		
Poorest	5,911 (24%)	31,012 (23%)
Poorer	5,286 (21%)	28,013 (21%)
Middle	4,874 (20%)	26,500 (20%)
Richer	4,584 (19%)	25,430 (19%)
Richest	4,015 (16%)	21,811 (16%)
Source of drinking water		
Not Improved	3,264 (13%)	9,818 (7.4%)
Improved	21,406 (87%)	122,949 (93%)
Sex of child		
Male	13,450 (55%)	72,466 (55%)
Female	11,220 (45%)	60,301 (45%)
Breastfed within1hr		
Yes	6,153 (25%)	58,447 (44%)
No	18,517 (75%)	74,320 (56%)
Complementary food in 3 days		
Given Nothing	10,642 (43%)	105,080 (79%)
Given something	14,028 (57%)	27,687 (21%)
Sex of head of household		
Male	21,886 (89%)	117,032 (88%)
Female	2,784 (11%)	15,735 (12%)
Comorbidities	6,440 (26%)	29,818 (22%)
Diet Diversity Score (6–23 months old child)		
0	1,274 (12%)	9,591 (16%)
1	2,421 (22%)	12,014 (21%)
2	3,394 (31%)	14,140 (24%)
3	2,347 (21%)	9,703 (17%)
4	1,083 (9.8%)	6,089 (10%)
5	414 (3.7%)	3,286 (5.6%)
6	121 (1.1%)	1,822 (3.1%)
7	21 (0.2%)	1,678 (2.9%)
Diet Diversity Score (6–23 months old child)^2^	2.00 (1.00, 3.00)	2.00 (1.00, 3.00)

^1^n (%); ^2^Median (25%,75%), NFHS-National Family Health Survey

**Table 2:** Association of per AFE intake of milk products intake with HAZ and WAZ

Dependent Variable	Independent Variable	^a^Unstandardized path coefficient (95% CI)	
		NFHS-3 (2004–2005)	NFHS-4 (2014–2015)
Path 1: Wealth Index to Per-AFE milk intake (in 10gm/day) to HAZ			
HAZ	Wealth Index	0.145 (0.129, 0.16)	0.102 (0.093, 0.11)
Milk and Milk Products	Wealth Index	3.338 (3.179, 3.494)	3.444 (3.375, 3.499)
HAZ	Milk and Milk Products	0.001 (0, 0.002)	0.002 (0.002, 0.003)
Path 2: Wealth Index to Per-AFE milk intake (in 10gm/day) to WAZ			
WAZ	Wealth Index	0.14 (0.125, 0.157)	0.096 (0.09, 0.102)
Milk and Milk Products	Wealth Index	3.379 (3.234, 3.512)	3.348 (3.286, 3.418)
WAZ	Milk and Milk Products	-0.001 (-0.002, 0)	-0.001 (-0.001, 0)

^a^Unstandardized path coefficient- Regression coefficient

Except for a few interior villages, the 68th round of the NSSO’s ninth quinquennial Household Consumer Expenditure survey, conducted during July 2011 to June 2012, covered all areas of India (29 states and 6 union territories, over 7469 villages and 5268 urban blocks). NSS 68 surveyed 59,683 rural households and 41,968 urban households. This survey collected monthly household consumer expenditure as well as household food purchase data of 223 food items for a 30-day recall period. Data on foods consumed from household production was also included. The seventh quinquennial consumer expenditure survey of the NSSO during the 61st round covering the period from June 2004 to July 2005 surveyed 79,298 households in rural India and 45,346 in urban India. The household’s food purchases were transformed into milk and milk product equivalents by referencing the Indian food composition Table [26]. Quantities listed by number or cost were standardized into food weights. Milk consumption per adult female equivalent (AFE) was derived by dividing the total household dairy intake by the count of AFEs in the household. AFEs were calculated as the ratio of each household member’s energy requirement to that of an adult female.

### Statistical matching

Non-Iterative Bayesian Approach to Statistical Matching (NIBAS) method was used to create two separate synthetic data sets constituting data on child anthropometric measures and nutrient intake at the household level for the periods of the two NFHS surveys. While NSS 68 was matched with NFHS-4, NSS 61 was statistically matched with NFHS-3. The matching process was specifically conducted within each state of residence to ensure the refinement and accuracy of the matched datasets. NFHS datasets were considered as the receiver dataset which received the missing milk and milk products intake data from the donor datasets, i.e., the NSS datasets. The matching variables considered were residence type (urban/rural), religion (Hindu/Muslim/Christian/others), caste (SC/ST/OBC/others), household size, cooking fuel (clean/not clean), sex of the household head(male/female),house ownership (own/not own), month of data collection(1 to 12), education of the head of the household(no education, primary, secondary, higher), as these were present in both the datasets and were associated with child anthropometric measurements.

The NIBAS approach is a full parametric Bayesian approach where a general linear model is assumed in both donor and receiver datasets and the non-computable full correlation matrix which is used for the computation of the variance parameter is obtained from an auxiliary dataset [27]. We considered data from a double fortified salt evaluation survey conducted in Uttar Pradesh (unpublished data) as the auxiliary data, where both anthropometry of children aged 6 to 59 months and child nutrient intake were jointly observed in 1200 children. The dietary data was collected using 24-hour recall method and the height measurements of children over the age of two years were carried out using a Seca stadiometer to an accuracy of 0.1 cm. Recumbent length of children under the age of two was measured with a portable Seca length board to an accuracy of 0.1 cm. Weight of children was measured using an electronic Seca scale (accuracy to 0.1 g).

Consider general linear models based on NFHS (receiver) and NSS (donor) datasets,

$$Y={X}_{A}{\beta }_{YX}+{U}_{A}, {U}_{A} \sim {N}_{pnA}\left({{\Sigma }}_{22}{I}_{nA}\right)\left(\text{u}\text{s}\text{i}\text{n}\text{g} \text{N}\text{F}\text{H}\text{S} \text{d}\text{a}\text{t}\text{a}\right)$$

$$Z={X}_{B}{\beta }_{ZX}+{U}_{B}, {U}_{B} \sim {N}_{qnB}\left({{\Sigma }}_{11}{I}_{nB}\right)\left(\text{u}\text{s}\text{i}\text{n}\text{g} \text{N}\text{S}\text{S} \text{D}\text{a}\text{t}\text{a}\right)$$

Where $Y$ is the HAZ/WAZ data present in the NFHS and $Z$ is the milk and milk products intake data present in the NSS dataset. ${X}_{A}$ and ${X}_{B}$ are the common variables present in NFHS and NSS respectively. Details of the NIBAS method is provided in Supplementary file 1.

### Path analysis

The conceptual framework (Fig. 1) presented in this study serves as a visual tool aimed at facilitating data analysis and guiding our statistical modeling. It primarily focuses on delineating potential pathways through which household wealth influences HAZ and WAZ among children aged 6 to 59 months, with a specific emphasis on the mediating role of per-AFE milk and milk product intake. The variables and the pathways in the framework were identified after a literature search in google scholar, PubMed databases and the search terms used were -stunting, underweight, association, Causal analysis, path analysis, for the period 2010–2021. There were 13 papers that exhaustively reported the factors associated with stunting and underweight. In addition, the UNICEF framework for stunting [28, 29] and expert advice were sought to revise the framework. The framework was the same for both stunting and underweight. The framework was used to examine the direct association of wealth and the indirect association mediated through per-AFE milk and milk product intake primarily, along with multiple other mediating variables on stunting and underweight (Fig. 1).

#### Dependent variables

HAZ and WAZ were treated as the dependent variables in separate models.

#### Independent variables

Wealth index (poorest, poorer, middle, richer, richest) of the household was considered as the independent variable.

#### Intermediate variables

The intermediate variables are specifically those factors situated in the pathway between the wealth index and HAZ/WAZ. These include residence type (urban, rural), source of drinking water (improved, not improved) and milk and milk products.

The covariates considered were maternal BMI, maternal height, maternal education (no education, primary, secondary and higher), sex of the child (male, female), initiation of early breastfeeding (no, yes), complementary feeding in first 3 days (no, yes) and comorbidities in last two weeks (fever or diarrhea or ARI symptoms in last two weeks as no/ yes). The variable “complementary food in 3 days” refers to foods or liquids other than breast milk that were given to infants within the first 3 days of their birth. It includes a range of items such as water, sugar/glucose water, fruit juice, infant formula, tea/infusions, honey, and more. This data was captured in the NFHS surveys. These variables may not be causally associated with household wealth but are relevant confounders due to their potential influence on child anthropometric outcomes and their association with household wealth. Detailed regression equations are given in Supplementary material.

To estimate the association of daily milk intake on HAZ and WAZ, linear regression models adjusted for other potential risk factors (variables specified in framework) were fitted.

Separate Path models were employed to examine the association of wealth quintiles on HAZ and WAZ. The total association between wealth and HAZ/WAZ was the sum of direct association between the variables and the indirect association between the variables through all intermediary variables. The overall association of wealth over HAZ/WAZ was decomposed into direct and indirect associations mediated through milk intake^2^ as well as maternal height and sanitation, where source of water was used as an indicator variable for sanitation. The model is explained below:

The final model is a linear model. Let Y be the matrix of all observed variables. The underlying model is a general linear model [30]:

$$Y=\text{Y}{\upbeta }+{\upepsilon }$$

Where the parametric matrix β is trimmed to include only paths of interest, paths specified in the conceptual framework. We have assumed ε are independent. Instead of minimizing the sum of squared errors, the free parameters are estimated using the sample covariance structure of the data. The covariance matrix from the above equation as

$$\sum ={Y}^{T}Y = {(1-{\upbeta })}^{-T}\left({{\upepsilon }}^{T}{\upepsilon }\right){(1-{\upbeta })}^{-1}$$

The sample covariance is:

$$S=\frac{1}{n-1}{Y}^{T}Y$$

Where n is the number of observations.

We have used the maximum likelihood (ML) method for estimation. With ML method the discrepancy function (F_min_) is given by the following equation;

$${F}_{ML}=\text{l}\text{o}\text{g}\left|\text{S}\right|-log|\sum | - \text{t}\text{r}\left(\text{S}{\sum }^{-1}\right)-\text{k}$$

where log represents the natural logarithm function with base ‘e,‘, || denotes the matrix determinant, k represents the number of variables within the correlation (or covariance) matrix, tr() is the trace of the matrix, S corresponds to the observed matrix, Σ stands for the covariance matrix.

All the bivariate variables are recoded as 0/1 and considered as numeric. All the exogenous ordinal variables are considered as numeric reflecting the order of the variables [31].In the hypothesized model in Fig. 1, there are four pathways from wealth index to HAZ/WAZ. The pathway through per-AFE milk intake is of interest in this study.

The direct association of one variable on another corresponds to the path coefficient that connects them, which is the regression coefficient, estimated using ML method. The indirect association of wealth index on HAZ through milk intake is the product of two path coefficients. As a result, the total association is the sum of the independent variable’s direct and/or indirect associations on the dependent variable.

Total Association = Direct Association + Indirect Association.

Separate models were executed for HAZ and WAZ using NFHS-3 and 4 to examine change in association over the decade. Thus, a total of four models were considered. Bootstrapping was used to test the statistical significance of the indirect associations. R software version 4.0.2 [32] was used for data preparation, analyses, and reporting using package lavaan v0.6-8 [31].

## Results

 The average HAZ and WAZ in NFHS-3 was − 1.9 ± 1.63 and − 1.8 ± 1.24 and that in NFHS-4 was − 1.5 ± 1.67 and − 1.5 ± 1.22. In both NFHS-3 and 4, 54% of the children were boys (Table 1). The proportion of children below 1 year (6 months to 1 year) was 15.45% and 15.15% in NFHS-3 and NFHS-4 respectively. The median (IQR) of HAZ and WAZ for children in the poorest wealth quintile was − 2.46 (-3.48, -1.42) and − 2.28 (-3.10, -1.53) in NFHS-3 and − 2.08 (-3.04, -1.06) and − 2.01 (-2.75, -1.26) in NFHS-4 and the median monotonically increased with increasing wealth quintile. (Supplementary file 1, Table 3). The overall diet diversity score for children aged less than 2 year was median = 2, (Q1-Q3: 1–3) in both NFHS-3 and NFHS-4. In children aged 6 to 23 m the diet diversity score was positively associated with HAZ and WAZ (Supplementary file 1, Tables 4 and 5) in both NFHS-3 and 4. The minimum dietary diversity score, which signifies the proportion of children aged 6–23 months consuming foods from four or more distinct food groups [33], was 15% for NFHS-3 and 22% for NFHS-4. The regression coefficients for the association of diversity score with HAZ in NFHS-3 and 4 was (${{\upbeta }}_{\text{N}\text{F}\text{H}\text{S}3}$=0.076, 95% CI: 0.04–0.09; ${{\upbeta }}_{\text{N}\text{F}\text{H}\text{S}4}$=0.03, 95% CI:0.02–0.04), and that of WAZ was (${{\upbeta }}_{\text{N}\text{F}\text{H}\text{S}3}$=0.05, 95% CI: 0.03–0.07; ${{\upbeta }}_{\text{N}\text{F}\text{H}\text{S}4}$=0.056, 95% CI:0.05–0.06) in children 6–23 m. But the association of diet diversity could not be examined in older children as this data is not available in the NFHS survey. All the sociodemographic characteristics were significantly associated with both HAZ and WAZ in multiple regression in both rounds of NFHS (Supplementary file 1, Tables 6 and 7).

 The intake of milk increased from NFHS-3 to NFHS-4, i.e.: the estimates of milk consumption based on the results predicted using NIBAS (Supplementary file 1 Table 8). The median per-AFE daily intake of milk and milk products were 88.96gm (Q1-Q3: 41.2-254.7) and 101.66 (Q1-Q3: 32.47–220.84) in NFHS-3 and NFHS-4 respectively.

### Path analysis

Wealth index was positively associated with both HAZ and WAZ in the path models for both NFHS-3 and 4 (Table 2). The direct association of wealth index on HAZ and WAZ reduced from NFHS-3 to NFHS-4. The regression coefficient of wealth index on HAZ in the path model for milk was ${{\upbeta }}_{ \text{W}\text{e}\text{a}\text{l}\text{t}\text{h}\text{N}\text{F}\text{H}\text{S}3}=$0.145 (95% CI: 0.129, 0.16) in NFHS-3 compared to ${{\upbeta }}_{ \text{W}\text{e}\text{a}\text{l}\text{t}\text{h}\text{N}\text{F}\text{H}\text{S}4}=$ 0.102 (0.093,0.11) in NFHS-4. This suggests that moving up one quintile in wealth index is associated with a 0.148 SDs in HAZ in NFHS-3, the effect size was smaller in NFHS-4. Wealth index was associated with per-AFE milk intake/day (intake in units of 10gm) in NFHS-3 and NFHS-4; (3.338; 95%: 3.179, 3.494) to NFHS-4 (3.444; 95%: 3.375, 3.499). This suggests that moving up one quintile in wealth index is associated with a 33.38 gm increase in per-AFE milk intake/day in NFHS-3 and 34.44 gm increase in NFHS4. In both NFHS-3 and 4, there was a small but positive association of per-AFE milk (*10 gm/day*) intake with HAZ (${{\upbeta }}_{ \text{N}\text{F}\text{H}\text{S}3}$=0.001;95% CI: 0, 0.002 and ${{\upbeta }}_{\text{N}\text{F}\text{H}\text{S}4}$=0.002;95% CI: 0.002, 0.003). This suggests that for every 10 gm per AFE increase in daily milk consumption, there is an estimated increase of approximately 0.001 in HAZ in NFHS-3 and 0.002 in HAZ in NFHS-4. The indirect association of wealth on HAZ incorporating milk intake in the pathway was lower than the estimated direct association (${{\upbeta }}_{ \text{I}\text{n}\text{d}\text{i}\text{r}\text{e}\text{c}\text{t}\text{W}\text{e}\text{a}\text{l}\text{t}\text{h}\text{N}\text{F}\text{H}\text{S}3}=0.004$;95% CI: 0.001, 0.008 ) and ${{\upbeta }}_{ \text{I}\text{n}\text{d}\text{i}\text{r}\text{e}\text{c}\text{t}\text{W}\text{e}\text{a}\text{l}\text{t}\text{h}\text{N}\text{F}\text{H}\text{S}4}$=0.007 ;95% CI: (0.005, 0.009) for NFHS-3 and 4, respectively.

However, per-AFE milk intake had no association with WAZ in both rounds of NFHS. In addition, higher wealth quintiles was associated with urban residence (${{\upbeta }}_{\text{N}\text{F}\text{H}\text{S}3}$=0.181; 95% CI: 0.177, 0.185) in NFHS-3 and (${{\upbeta }}_{\text{N}\text{F}\text{H}\text{S}4}$=0.144; 95% CI: 0.142, 0.145) in NFHS-4. This means moving up one quintile in wealth was associated with 0.181 increase in the probability urban residence. Also, households in urban areas have access to improved source of drinking water (${{\upbeta }}_{\text{N}\text{F}\text{H}\text{S}3}$=0.139;95% CI: 0.127, 0.148) and (${{\upbeta }}_{\text{N}\text{F}\text{H}\text{S}4}$=0.062;95% CI: 0.059, 0.065) for NFHS-3 and 4.

 Children of mothers with higher levels of education were more likely to breastfeed within one hour of birth. Mother’s education was positively associated with HAZ. Breastfeeding within one hour of birth was also positively associated with HAZ. Complementary feeding within the first three days had a negative association with HAZ. Maternal height, BMI were all positively associated with HAZ in both rounds and access to drinking water was negatively associated with HAZ and WAZ in NFHS-3 and NFHS-4 (Supplementary file 1 Tables 9 and 10). Girl children had higher HAZ in both rounds.

 While the study primarily utilized continuous HAZ and WAZ as outcomes, it is important to note that HAZ and WAZ can also indicate being stunted and underweight when low. To explore this further, we conducted an analysis using binary HAZ and WAZ, categorizing children as underweight (WAZ < -2) or not (WAZ >= -2) and stunted (HAZ < -2) or not (HAZ >= -2). In NFHS-3 and 4, this analysis revealed no significant association of milk and milk Products with stunting or underweight (Supplementary file 1 Tables 11 and 12).

### Sensitivity analysis

To quantify the uncertainty in the matching process, a 10-iteration multiple imputation approach was used. Thus 10 triangulated datasets were generated using NIBAS. Path models were run using the 10 datasets and the estimates were compared. Path models using the 10 datasets provided similar results. The mean estimate of the effect of per-AFE milk intake on HAZ across the 10 models was 0.00093(standard error, SE: 0.0004) in NFHS-3 and 0.001 (SE: 0.0002) in NFHS-4, indicating a high degree of similarity between the models supporting the reliability of the matching process. (Supplementary file 2).

Additionally in a subset of households with children 6-23 m, a comparative analysis examining the AFE milk intake (from the statistically matched data of NSSO) with number of dairy products reported to be consumed by the child aged 6-23 m in the diet diversity section of the NFHS questionnaire was conducted to validate the NIBAS model. There was an increase in quantity of milk and milk product consumption (estimated using NIBAS) across the number (ranging from zero to 3 or more) of dairy products consumed in a day, reflecting strong agreement between estimated and reported dairy consumption values. In NFHS3, the NIBAS model-estimated median intake of dairy consumption was 59gm (IQR 15, 152), 106gm (IQR 34, 218), 149gm (IQR 56, 278) and 181gm (IQR 83, 345) for households reporting no dairy product consumption, one, two and three or more products, p-value < 0.001 by Kruskal-Wallis test). Similarly, in NFHS4 the quantities were 80gm (IQR 23, 183), 120gm (IQR 43, 241), 137gm (IQR 53, 271) and 142gm (IQR 53, 262), p-value < 0.001 (Supplementary Table 13).

## Discussion

The study examined the direct and indirect (through diet) association of household wealth index on anthropometric indices (HAZ and WAZ) in Indian children aged 6–59 months using two rounds of nationally representative NFHS datasets [20, 21]. As a direct association, the household wealth index was associated with 0.14 and 0.1 SD increase in HAZ and WAZ in NFHS-3 and 4, respectively. Among the sociodemographic factors, households with access to improved source of drinking water had lower HAZ and WAZ scores. Earlier studies [34, 35] also reported such findings, however, possible explanation for this counter intuitive association is not available. Studies from several cross-sectional and longitudinal studies, particularly from LMICs have previously demonstrated that adverse socioeconomic conditions and poverty in early life influences physical growth in children [36–41]. An analysis using data from eight longitudinal cohorts in four countries i.e., Ethiopia, India, Peru, and Vietnam, showed that household wealth index was significantly associated with increased HAZ with co-efficient ranging from 0.01 to 0.021 across the countries [42]. However, none of these studies had investigated the role of diet in the association between wealth index and anthropometric outcomes.

Inadequate dietary intake is one of the immediate underlying causes of child growth faltering and in turn stunting [16, 43, 44]. The dietary nutrient adequacy can be evaluated by computing a diet diversity score, which also associates with linear growth and other nutritional outcomes in children [7, 43]. The minimum dietary diversity (MDD) is one such score, which is dependent on the frequency of consumption of food groups but not based on quantitative evaluation of those food groups. A recent analysis using Demographic Health Survey data from 39 countries (n = 74,548) showed that children aged 6–23 months with minimum diversity in their diets had 1.345 times higher odds of being stunted when compared to consuming diets with optimal diversity; higher odds of being stunted (1.436 times) was observed for those who did not consume any animal source foods [43]. Such associations were more pronounced in children from LMICs with a higher risk of stunting (OR: 1.42 times) compared to children from high income counties (OR: 1.25 times) [43]. However, use of scores such as MDD as proxy indicators to assess nutrient adequacy could be confounding with limited diversity in diets, as commonly observed in < 2y old children, who are predominantly breastfed and are often not introduced to optimal complementary feeding practices. This is supported by the recent reports, showing that an adequate diet could explain only 9% [19] to 13% variability of stunting in 6–23 m old children [43], and highlighting the need for the better-quality dietary intake data to study its association with stunting in aged 6–59 months children. This is partly because diet diversity scores do not consider the quantity of foods consumed. Children with very little consumption of the food group are grouped along with those who consume sufficiently and the variability in consumption is not adequately captured in these scores.

Several observational and intervention studies have shown positive association between animal source foods, particularly dairy products and growth outcomes in children [15, 45–48]. A recent analysis of data from Chinese Nutrition and Health Surveillance demonstrated that HAZ for children who consumed milk and milk products at least once per day or per week was 0.11 points or 0.13 points higher than the children without dairy intake. The risk of stunting was 28% lower in children consuming milk and milk products at least once in a day compared to absence of intakes in the last week [45]. The growth stimulating association of milk could partly be attributed to the increased expression of circulating insulin-like growth factor upon intake [48]. Additionally, when compared to other animal source foods (such as eggs and meat), milk has higher content of calcium and Vitamin D, which is supported by their independent associations on linear growth and stunting [48, 49]. With the limited experimental evidence from less developed settings, ecological analyses and observational studies reported that consumption of milk and milk products were associated with increased HAZ score in children of < 5 y [50–52].

Due to the lacto-vegetarian predominance (34% of population), milk and milk products serve as high quality protein source and the growth promoting micronutrients (such as Vitamin B_12_, Vitamin D, calcium and zinc) in Indian diets [53, 54]. The present study showed that milk and milk products had a significant modifying association (i.e., indirect association) in the association between wealth index and HAZ in NFHS-3 (β: 0.004) and NFHS-4 (β: 0.007) survey; the association being pronounced (2-fold higher) in the latter and was indicative of the economic growth and its positive impact on dairy consumption overtime [55]. A recent analysis using data from mini demographic and health survey in Ethiopian children showed that the socioeconomic, biological, and behavioral characteristics of the child, mother, and household could explain about two third of the total association of wealth on stunting [56]; however, none of the previous studies have investigated the modifying association of ASF consumption on anthropometric indices. Additionally, residence, sex of the child, place of delivery, use of contraceptives, mothers’ education level, and family size were important determinants of stunting, and this was in line with other studies from developing countries [57–59].

This is one of the few studies assessing the modifying association of milk and milk product consumption on the association between wealth and anthropometric indices, which used a conceptual framework approach to account for the confounding covariates. The study had few limitations. In absence of a single nationally representative survey that simultaneously collected data on food intakes and anthropometry, statistical matching between 2 major national-level survey datasets i.e., NFHS and NSSO was conducted using the NIBAS technique to obtain a robust dataset containing data on both dietary intake and anthropometric indices. This matching of NFHS and NSS data was facilitated by using an auxiliary dataset from a double fortified salt evaluation survey (unpublished data), which simultaneously collected data on dietary intakes and anthropometry parameters using precise methods. The quantitative consumption of milk and milk products from NIBAS increased with higher frequency of consumption as reported in NFHS. It is important to note that the data collection years for the NHFS-4 survey did not align with the dates for the NSS-68th round. There was a mean temporal difference of 3 years between both the surveys. As a result, socioeconomic, climatic, and other conditions may have differed between the two periods of data collection. Furthermore, we were unable to use the latest round of NFHS and NSS data due to the unavailability of contemporary NSS data. Also, the milk consumption data used in this study were at the household level per AFE, rather than at the level of the individual child. This distinction is significant as it may not fully capture individual-level variations. Breastmilk intake by the child was not considered in this analysis as the data was not available. Additionally, there was a possibility of reverse causation between exogenous measure of wealth and other predictors of growth faltering with anthropometric indices, which was not considered in the present analyses. In conclusion, this study contributes insights about the salient and persistent associations between wealth and early life physical growth and their interaction with dairy consumption and presents a statistical matching approach to examining data when relevant exposures and outcomes are not captured in a single survey. The work also suggests the potential role of including milk and milk products in the dietary interventions and supplementary nutrition programs to combat undernutrition in LMICs.

### Supplementary Information

**Additional file 1: Supplementary Fig. 1(a):** Flowchart for selection children for analysis from NFHS-4 dataset. **Supplementary Fig. 1(b):** Flowchart for selection children for analysis from NFHS-3 dataset. **Supplementary Table 1: **Comparison of Demographic Characteristics between Excluded and Final Analytical Data- NFHS-3. **Supplementary Table 2:** Comparison of Demographic Characteristics between Excluded and Final Analytical Data- NFHS-4.**Supplementary Table 3:** Distribution of Weight for Age Z score (WAZ) and Height for age Z score (HAZ) across sociodemographic characteristic. **Supplementary Table 4**: Multiple linear regression of HAZ for children aged 6- 23 months. **Supplementary Table 5:** Multiple linear regression of WAZ for children aged 6- 23 months. **Supplementary Table 6:** Multiple linear regression on HAZ for all children aged 6 to 59m .**Supplementary Table 7:** Multiple linear regression on WAZ for all children aged 6m to 59 months. **Supplementary Table 8**: Per-consumption unit (adult female equivalent) intake of milk& milk products in triangulated data of NFHS-3 and NFHS-4. **Supplementary Table 9:** Path analysis coefficients of HAZ. **Supplementary Table 10:** Path analysis coefficients of WAZ. **Supplementary Table 11: **Path analysis coefficients of Underweight (WAZ < -2 vs WAZ>=-2). **Supplementary Table 12: **Path analysis coefficients of Stunting (HAZ < -2 vs HAZ >=-2). **Supplementary Table 13:** Comparison of milk intake (Statistically matched by NIBAS) with consumption of dairy products for children aged 6-23 months.

**Additional file 2: Supplementary file 2.** Results from multiple imputation for WAZ (NFHS3).

## Data Availability

The datasets used in this study are available in the public domain. The National Family Health Survey (NFHS) datasets can be obtained from the Demographic and Health Surveys (DHS) program website (https://dhsprogram.com/data/available-datasets.cfm). The National Sample Survey (NSS) datasets can be downloaded from the official website of the Ministry of Statistics and Programme Implementation, Government of India (http://mospi.gov.in/national-sample-survey-nss). The unpublished data from the double fortified salt evaluation survey conducted in Uttar Pradesh is available from the corresponding author on reasonable request.

## Abbreviations

ASF: Animal source foods
HAZ: Height for age
WAZ: Weight for age
NFHS: National Family Health Survey
NSS: National Sample Survey
NIBAS: Non-Iterative Bayesian Approach to Statistical Matching
