# Supplementary material for: The role of dairy consumption in the relationship between wealth and early life physical growth in India: evidence from multiple national surveys
Source: BMC Public Health. 2024 Jan 5;24:96. doi: 10.1186/s12889-023-17520-8 (PMC10768164; doi:10.1186/s12889-023-17520-8)
Supplement: Supplementary file 1 — Additional file 1: Supplementary Fig. 1(a): Flowchart for selection children for analysis from NFHS-4 dataset. Supplementary Fig. 1(b): Flowchart for selection children for analysis from NFHS-3 dataset. Supplementary Table 1: Comparison of Demographic Characteristics between Excluded and Final Analytical Data- NFHS-3. Supplementary Table 2: Comparison of Demographic Characteristics between Excluded and Final Analytical Data- NFHS-4.Supplementary Table 3: Distribution of Weight for Age Z score (WAZ) and Height for age Z score (HAZ) across sociodemographic characteristic. Supplementary Table 4: Multiple linear regression of HAZ for children aged 6- 23 months. Supplementary Table 5: Multiple linear regression of WAZ for children aged 6- 23 months. Supplementary Table 6: Multiple linear regression on HAZ for all children aged 6 to 59m .Supplementary Table 7: Multiple linear regression on WAZ for all children aged 6m to 59 months. Supplementary Table 8: Per-consumption unit (adult female equivalent) intake of milk& milk products in triangulated data of NFHS-3 and NFHS-4. Supplementary Table 9: Path analysis coefficients of HAZ. Supplementary Table 10: Path analysis coefficients of WAZ. Supplementary Table 11: Path analysis coefficients of Underweight (WAZ < -2 vs WAZ>=-2). Supplementary Table 12: Path analysis coefficients of Stunting (HAZ < -2 vs HAZ >=-2). Supplementary Table 13: Comparison of milk intake (Statistically matched by NIBAS) with consumption of dairy products for children aged 6-23 months. [file 12889_2023_17520_MOESM1_ESM.docx]

**Supplementary material**

**Details of the NIBAS method**

Consider general linear models based on NFHS (receiver) and NSS (donor) datasets,

$$Y=X_{A}\beta_{YX}+U_{A}, U_{A} \sim N_{pnA}(\Sigma_{22}I_{nA})(using NFHS data)$$

$$Z=X_{B}\beta_{ZX}+U_{B}, U_{B} \sim N_{qnB}(\Sigma_{11}I_{nB})(using NSS Data)$$

Where $Y$ is the HAZ/WAZ data present in the NFHS and $Z$ is the milk and milk products intake data present in the NSS dataset. $X_{A}$ and $X_{B}$ are the common variables present in NFHS and NSS respectively. $\left[ \begin{matrix} \Sigma_{11} & \Sigma_{12} \\ \Sigma_{21} & \Sigma_{22} \end{matrix} \right]$ is the variance covariance matrix of the joint vector $[Z, Y]$. $U_{A}$ and $U_{B}$ are the error terms in the linear relationship. $\Sigma_{12}$ and $\Sigma_{21}$ are derived from a completely observed auxiliary dataset.

**Algorithm for NIBAS:**

- Compute the estimates for the parameters $\hat{\beta}_{YX}={({X^{'}}_{A}X_{A})}^{-1}{X^{'}}_{A}Y$ and $\hat{\beta}_{ZX}={({X^{'}}_{B}X_{B})}^{-1}{X^{'}}_{B}Z$ from the regression of each dataset.
- Calculate the following matrices proportional to the sample covariances for each regression with $S_{Y}=(Y- X_{A}\hat{\beta}_{YX})'(Y- X_{A}\hat{\beta}_{YX})$ and $S_{Z}=(Z- X_{B}\hat{\beta}_{ZX})'(Z- X_{B}\hat{\beta}_{ZX})$
- Choose a value for the correlation matrix $R_{YZ| X}$from the completely observed UP dataset.
- Perform random draws for the parameters from their observed-data posterior distribution according to the following scheme:

Step 1:

$\Sigma_{22/Y} \sim W_{p}^{-1}(v_{A}, S_{Y}^{-1})$ with $v_{A}=n_{A}-\left( k+p \right)+1$

$\Sigma_{11/Z} \sim W_{q}^{-1}(v_{B}, S_{Z}^{-1})$ with $v_{B}=n_{B}-\left( k+q \right)+1$

Step 2:

$\hat{\beta}_{YX}/\Sigma_{22},y \sim N_{pk}(\hat{\beta}_{YX},\Sigma_{22}{(X_{A}'X_{A})}^{-1} )$, $\hat{\beta}_{ZX}/\Sigma_{11},z \sim N_{qk}(\hat{\beta}_{ZX},\Sigma_{11}{(X_{B}'X_{B})}^{-1} ).$

Step 3:

Set $\Sigma_{12}=\{\sigma_{Y_{i}, Z_{j}/X}\}$ with $\sigma_{Y_{i}, Z_{j}/X}=\rho_{YiZj | X}\sqrt{{\sigma^{2}}_{Y_{i}/X}{\sigma^{2}}_{Z_{j}/X}}$, With ${\sigma^{2}}_{Y_{i}/X}$ and ${\sigma^{2}}_{Z_{j}/X}$ derived in step 1 for $i=1, 2,\ldots, q, j=1, 2,\ldots, p$

Step 4:

$Y/z,\beta,\Sigma\sim N_{qnA}({Z_{A}\beta}_{YX},(Z- {X_{A}\beta}_{ZX}){\Sigma_{22}}^{-1} \Sigma_{21})$; ($\Sigma_{11}- \Sigma_{12}{\Sigma_{22}}^{-1}\Sigma_{21}$)$I_{nA}$

Repeating this procedure m times yields m imputed datasets which would be analysed by standard complete data inference. The results are combined then according to the MI paradigm.

**Regression Equations of Path Analysis:**

The regression equations that constituted the path model are given below:

- Residence Type=β_1_​×Wealth Index+ ε_1_
- Source of drinking water= β_2_×Residence Type+ ε_2_
- Milk & Milk Products = β_3_Wealth Index+ ε_3_
- Breastfed within 1hr= β_4_×Mother’s Education+ ε_4_
- Complementary food in 3 days= β_5_×Mother’s Education+ ε_5_
- HAZ = β_6_×Source of drinking water + β_7_×Complementary food in 3 days + β_8_×Breastfed within 1hr + β_9_×Mother's Education + β_10_×Wealth Index+ β_11_×Milk & Milk Products + β_12_×Mother's Height+ β_13_×Mother's BMI + β_14_×Sex of child + β_15_×Comorbidities+ ε_6_

**Supplementary Fig. 1(a):** Flowchart for selection children for analysis from NFHS-4 dataset.

NFHS-4 children aged <59 and >6 months **221858**

259627

**15785**- Excluded children with missing values for HAZ or WAZ

Excluded because of missing data in source of drinking water/complimentary food in 3 days /breastfeeding within 1 hour- **69406**

Excluded due to outlier values for mother’s BMI (<=12 or >=50 kg/m^2^)-**790 a7790jjdddsdd7790**790

Final Analysis

**132767**

Energy Outliers (<500 Kcal & >5000Kcal) - **482**

Excluded due to outlier values for mother’s height (<120 or >180 cm)**- 253**

**Excluded 530** - Andaman and nicobar islands

**160**– Chandigarh

**252**- Dadra and nagar haveli

**302** - Daman and diu

**250** - Lakshadweep

**881** – Puducherry

**Supplementary Fig. 1(b):** Flowchart for selection children for analysis from NFHS-3 dataset.

NFHS-3 Children aged <59 and >6 months **43419**

**Missing Values**

**12984**– Excluded because of missing observations in source of drinking water / complimentary food in 3 days / breastfeeding within 1 hour

**5459** – Excluded children with missing values for HAZ or WAZ

**238**– Excluded outlier values for mothers BMI (<=12 or >=50)

Final Analysis

**24670**

Energy Outliers(<500 Kcal & >5000Kcal) - **64**

**4**- Excluded outlier values for mothers Height (<120 or >180)

**Supplementary Table 1:** Comparison of Demographic Characteristics between Excluded and Final Analytical Data- NFHS-3

|  | **Excluded from Analysis, N = 18,749** | | **Included in Analysis, N = 24,670** | |
| --- | --- | --- | --- | --- |
| **Characteristic** | **n (%)** | **95% CI** | **n (%)** | **95% CI** |
| Child's age in months, Mean (SD) | 38 (14) | 37.7, 38.1 | 28 (15) | 28.0, 28.4 |
| Place of Residence |  |  |  |  |
| Urban | 7,079 (38%) | 37, 38 | 9,620 (39%) | 38, 40 |
| Rural | 11,670 (62%) | 62, 63 | 15,050 (61%) | 60, 62 |
| Household Wealth Quintiles |  |  |  |  |
| Poorest | 3,454 (18%) | 18, 19 | 4,068 (16%) | 16, 17 |
| Poorer | 3,606 (19%) | 19, 20 | 4,237 (17%) | 17, 18 |
| Middle | 3,924 (21%) | 20, 22 | 5,005 (20%) | 20, 21 |
| Richer | 4,115 (22%) | 21, 23 | 5,589 (23%) | 22, 23 |
| Richest | 3,650 (19%) | 19, 20 | 5,771 (23%) | 23, 24 |
| Sex of child |  |  |  |  |
| Male | 9,306 (50%) | 49, 50 | 13,394 (54%) | 54, 55 |
| Female | 9,443 (50%) | 50, 51 | 11,276 (46%) | 45, 46 |

**Supplementary Table 2:** Comparison of Demographic Characteristics between Excluded and Final Analytical Data- NFHS-4

|  | **Excluded From Analysis, N = 89,091** | | **Included In Analysis, N = 132,767** | |
| --- | --- | --- | --- | --- |
| **Characteristic** | **n (%)** | **95% CI** | **n (%)** | **95% CI** |
| Child's age in months, Mean(SD) | 38 (14) | 38.3, 38.5 | 28 (15) | 28.3, 28.4 |
| Place of Residence |  |  |  |  |
| Urban | 20,350 (23%) | 23, 23 | 33,043 (25%) | 25, 25 |
| Rural | 68,741 (77%) | 77, 77 | 99,724 (75%) | 75, 75 |
| Household Wealth Quintiles |  |  |  |  |
| Poorest | 25,431 (29%) | 28, 29 | 32,382 (24%) | 24, 25 |
| Poorer | 21,527 (24%) | 24, 24 | 30,497 (23%) | 23, 23 |
| Middle | 17,456 (20%) | 19, 20 | 26,904 (20%) | 20, 20 |
| Richer | 14,157 (16%) | 16, 16 | 23,108 (17%) | 17, 18 |
| Richest | 10,520 (12%) | 12, 12 | 19,876 (15%) | 15, 15 |
| Sex of child |  |  |  |  |
| Male | 43,060 (48%) | 48, 49 | 72,294 (54%) | 54, 55 |
| Female | 46,031 (52%) | 51, 52 | 60,473 (46%) | 45, 46 |

## **Supplementary Table 3:** Distribution of Weight for Age Z score (WAZ) and Height for age Z score (HAZ) across sociodemographic characteristic

|  | **WAZ** | | **HAZ** | |
| --- | --- | --- | --- | --- |
| **Characteristic** | **NFHS-3** | **NFHS-4** | **NFHS-3** | **NFHS-4** |
| Age in Years |  |  |  |  |
| <1 Year | -1.55 (-2.41, -0.68) | -1.33 (-2.15, -0.51) | -1.13 (-2.15, -0.10) | -0.81 (-1.90,0.29) |
| 1-3 Year | -1.86 (-2.68, -1.03) | -1.61 (-2.38, -0.80) | -2.17 (-3.12, -1.14) | -1.73 (-2.70, -0.70) |
| 3-5 Year | -1.84 (-2.58, -1.06) | -1.64 (-2.37, -0.91) | -1.98 (-2.91, -1.02) | -1.64 (-2.47, -0.75) |
| Mother's BMI |  |  |  |  |
| <18.5 | -2.08 (-2.89, -1.33) | -1.96 (-2.68, -1.20) | -2.14 (-3.11, -1.15) | -1.85 (-2.79, -0.85) |
| 18.5-24.9 | -1.69 (-2.48, -0.86) | -1.54 (-2.30, -0.77) | -1.89 (-2.89, -0.86) | -1.56 (-2.52, -0.52) |
| >=25 | -1.06 (-1.83, -0.35) | -1.16 (-1.88, -0.37) | -1.28 (-2.20, -0.40) | -1.22 (-2.12, -0.27) |
| Residence Type |  |  |  |  |
| Urban | -1.45 (-2.29, -0.68) | -1.35 (-2.13, -0.53) | -1.59 (-2.59, -0.59) | -1.30 (-2.24, -0.28) |
| Rural | -1.92 (-2.72, -1.12) | -1.67 (-2.43, -0.90) | -2.08 (-3.05, -1.06) | -1.68 (-2.64, -0.67) |
| Mother's Education |  |  |  |  |
| No Education | -2.12 (-2.97, -1.32) | -1.95 (-2.70, -1.20) | -2.33 (-3.36, -1.27) | -2.05 (-3.00, -1.01) |
| Primary | -1.83 (-2.60, -1.11) | -1.72 (-2.45, -0.98) | -2.01 (-2.91, -1.08) | -1.80 (-2.67, -0.83) |
| Secondary | -1.51 (-2.23, -0.71) | -1.45 (-2.20, -0.69) | -1.61 (-2.51, -0.67) | -1.41 (-2.31, -0.43) |
| Higher | -0.84 (-1.66, -0.14) | -0.99 (-1.77, -0.21) | -0.93 (-1.79, -0.01) | -0.90 (-1.85,0.04) |
| Wealth Index |  |  |  |  |
| Poorest | -2.28 (-3.10, -1.53) | -2.01 (-2.75, -1.26) | -2.46 (-3.48, -1.42) | -2.08 (-3.04, -1.06) |
| Poorer | -2.03 (-2.83, -1.25) | -1.75 (-2.50, -1.02) | -2.19 (-3.15, -1.19) | -1.80 (-2.71, -0.81) |
| Middle | -1.80 (-2.55, -1.05) | -1.54 (-2.26, -0.80) | -2.01 (-2.92, -1.05) | -1.55 (-2.44, -0.56) |
| Richer | -1.54 (-2.27, -0.78) | -1.33 (-2.08, -0.55) | -1.71 (-2.62, -0.74) | -1.30 (-2.19, -0.30) |
| Richest | -1.07 (-1.85, -0.32) | -1.06 (-1.80, -0.26) | -1.14 (-2.06, -0.28) | -0.98 (-1.90, -0.01) |
| Source of drinking water |  |  |  |  |
| Not Improved | -1.98 (-2.77, -1.11) | -1.68 (-2.46, -0.92) | -2.02 (-3.08, -0.97) | -1.63 (-2.62, -0.57) |
| Improved | -1.78 (-2.58, -0.97) | -1.57 (-2.34, -0.78) | -1.95 (-2.92, -0.91) | -1.57 (-2.52, -0.54) |
| Sex |  |  |  |  |
| Male | -1.79 (-2.60, -0.97) | -1.60 (-2.37, -0.83) | -1.98 (-2.97, -0.95) | -1.61 (-2.57, -0.59) |
| Female | -1.82 (-2.62, -0.99) | -1.55 (-2.33, -0.75) | -1.93 (-2.90, -0.89) | -1.53 (-2.48, -0.49) |
| BF within1hr^1^ |  |  |  |  |
| Yes | -1.61 (-2.40, -0.82) | -1.55 (-2.31, -0.75) | -1.76 (-2.72, -0.77) | -1.52 (-2.46, -0.49) |
| No | -1.87 (-2.67, -1.05) | -1.60 (-2.37, -0.82) | -2.02 (-3.01, -0.98) | -1.62 (-2.58, -0.59) |
| Complementary food in 3 days^2^ |  |  |  |  |
| Given Nothing | -1.69 (-2.46, -0.86) | -1.58 (-2.35, -0.79) | -1.78 (-2.73, -0.76) | -1.56 (-2.52, -0.53) |
| Given something | -1.90 (-2.72, -1.10) | -1.58 (-2.34, -0.81) | -2.09 (-3.10, -1.05) | -1.63 (-2.57, -0.61) |
| Sex of head |  |  |  |  |
| Male | -1.80 (-2.60, -0.98) | -1.57 (-2.34, -0.79) | -1.95 (-2.93, -0.93) | -1.56 (-2.52, -0.54) |
| Female | -1.85 (-2.67, -1.05) | -1.62 (-2.38, -0.83) | -2.00 (-2.97, -0.89) | -1.66 (-2.61, -0.59) |
| Comorbidities |  |  |  |  |
| No | -1.78 (-2.59, -0.95) | -1.56 (-2.33, -0.77) | -1.96 (-2.95, -0.91) | -1.56 (-2.52, -0.54) |
| Yes | -1.89 (-2.66, -1.09) | -1.63 (-2.41, -0.86) | -1.95 (-2.91, -0.95) | -1.61 (-2.56, -0.57) |
| Diet Diversity Score^3^ |  |  |  |  |
| 0 | -1.84 (-2.71, -1.00) | -1.52 (-2.38, -0.73) | -1.61 (-2.61, -0.52) | -1.20 (-2.40, -0.05) |
| 1 | -1.84 (-2.67, -0.94) | -1.54 (-2.37, -0.68) | -1.76 (-2.85, -0.70) | -1.38 (-2.49, -0.16) |
| 2 | -1.83 (-2.68, -0.97) | -1.51 (-2.31, -0.71) | -1.89 (-2.98, -0.74) | -1.47 (-2.57, -0.33) |
| 3 | -1.62 (-2.42, -0.78) | -1.52 (-2.30, -0.71) | -1.91 (-2.83, -0.82) | -1.56 (-2.61, -0.47) |
| 4 | -1.44 (-2.23, -0.64) | -1.39 (-2.16, -0.54) | -1.71 (-2.66, -0.64) | -1.44 (-2.48, -0.37) |
| 5 | -1.46 (-2.11, -0.51) | -1.33 (-2.11, -0.56) | -1.54 (-2.48, -0.61) | -1.48 (-2.48, -0.34) |
| 6 | -1.10 (-1.99, -0.39) | -1.37 (-2.08, -0.54) | -1.21 (-2.14, -0.52) | -1.42 (-2.46, -0.31) |
| 7 | -1.40 (-2.38,0.23) | -1.22 (-1.96, -0.43) | -0.93 (-2.75,0.75) | -1.40 (-2.39, -0.10) |
| Values presented are mean (95% Confidence Interval  ^1^Breastfeed within one hour of birth; ^2^Refers to foods or liquids other than breast milk that were given to infants within the first 3 days of their birth; ^3^Diet diversity score of children aged 6 - 23 months | | | | |

## **Supplementary Table 4**: Multiple linear regression of HAZ for children aged 6- 23 months

|  | **NFHS-3** | | **NFHS-4** | |
| --- | --- | --- | --- | --- |
| **Characteristic** | **Regression Coefficient** | **95% CI** | **Regression Coefficient** | **95% CI** |
| Age in Years |  |  |  |  |
| 6-11 Month | — | — | — | — |
| 11.1-23 Month | -1.0 | -1.0, -0.89 | -0.85 | -0.88, -0.82 |
| Residence Type |  |  |  |  |
| Urban | — | — | — | — |
| Rural | 0.08 | 0.01, 0.16 | 0.03 | -0.01, 0.07 |
| Mother's Education |  |  |  |  |
| No Education | — | — | — | — |
| Primary | 0.11 | 0.01, 0.21 | 0.10 | 0.05, 0.14 |
| Secondary | 0.27 | 0.18, 0.35 | 0.27 | 0.24, 0.31 |
| Higher | 0.59 | 0.45, 0.73 | 0.49 | 0.43, 0.55 |
| Wealth Index |  |  |  |  |
| Poorest | — | — | — | — |
| Poorer | 0.22 | 0.12, 0.32 | 0.19 | 0.15, 0.23 |
| Middle | 0.35 | 0.24, 0.45 | 0.40 | 0.36, 0.45 |
| Richer | 0.57 | 0.45, 0.68 | 0.57 | 0.52, 0.62 |
| Richest | 0.90 | 0.76, 1.0 | 0.77 | 0.71, 0.83 |
| Source of drinking water |  |  |  |  |
| Not Improved | — | — | — | — |
| Improved | -0.18 | -0.26, -0.10 | -0.1 | -0.17, -0.07 |
| Sex of child |  |  |  |  |
| Male | — | — | — | — |
| Female | 0.15 | 0.09, 0.22 | 0.21 | 0.19, 0.24 |
| Breastfed within1hr^1^ |  |  |  |  |
| Yes | — | — | — | — |
| No | -0.03 | -0.10, 0.05 | -0.05 | -0.08, -0.02 |
| Complementary food in 3 days^2^ |  |  |  |  |
| Given Nothing | — | — | — | — |
| Given something | -0.05 | -0.11, 0.02 | -0.04 | -0.07, 0.00 |
| Sex of head of household |  |  |  |  |
| Male | — | — | — | — |
| Female | 0.05 | -0.05, 0.15 | -0.02 | -0.07, 0.02 |
| Comorbidities |  |  |  |  |
| No | — | — | — | — |
| Yes | 0.02 | -0.04, 0.09 | -0.03 | -0.06, 0.00 |
| Diet Diversity Score^3^ | 0.07 | 0.04, 0.09 | 0.03 | 0.02, 0.04 |

CI= Confidence Interval; ^1^Breastfeed within one hour of birth; ^2^Refers to foods or liquids other than breast milk that were given to infants within the first 3 days of their birth; ^3^Diet diversity score of children aged 6 - 23 months

## **Supplementary Table 5:** Multiple linear regression of WAZ for children aged 6- 23 months

|  | NFHS-3 | | | NFHS-4 | |
| --- | --- | --- | --- | --- | --- |
| Characteristic | Regression Coefficient | 95% CI | Regression Coefficient | | 95% CI |
| Age in Years |  |  |  | |  |
| 6-11 Month | — | — | — | | — |
| 11.1-23 Month | -0.2 | -0.34, -0.24 | -0.28 | | -0.31, -0.26 |
| Residence Type |  |  |  | |  |
| Urban | — | — | — | | — |
| Rural | 0.03 | -0.02, 0.09 | 0.06 | | 0.03, 0.08 |
| Mother's Education |  |  |  | |  |
| No Education | — | — | — | | — |
| Primary | 0.17 | 0.10, 0.24 | 0.11 | | 0.08, 0.15 |
| Secondary | 0.3 | 0.25, 0.37 | 0.24 | | 0.21, 0.27 |
| Higher | 0.58 | 0.48, 0.69 | 0.45 | | 0.41, 0.49 |
| Wealth Index |  |  |  | |  |
| Poorest | — | — | — | | — |
| Poorer | 0.26 | 0.19, 0.34 | 0.25 | | 0.22, 0.28 |
| Middle | 0.48 | 0.40, 0.56 | 0.45 | | 0.41, 0.48 |
| Richer | 0.6 | 0.58, 0.75 | 0.63 | | 0.59, 0.67 |
| Richest | 0.94 | 0.84, 1.0 | 0.80 | | 0.76, 0.84 |
| Source of drinking water |  |  |  | |  |
| Not Improved | — | — | — | | — |
| Improved | -0.07 | -0.13, -0.01 | -0.13 | | -0.16, -0.09 |
| Sex of child |  |  |  | |  |
| Male | — | — | — | | — |
| Female | 0.07 | 0.03, 0.12 | 0.13 | | 0.11, 0.15 |
| Breastfed within1hr^1^ |  |  |  | |  |
| Yes | — | — | — | | — |
| No | -0.09 | -0.15, -0.04 | -0.09 | | -0.11, -0.07 |
| Complementary food in 3 days^2^ |  |  |  | |  |
| Given Nothing | — | — | — | | — |
| Given something | -0.02 | -0.07, 0.03 | 0.01 | | -0.01, 0.04 |
| Sex of head of household |  |  |  | |  |
| Male | — | — | — | | — |
| Female | 0 | -0.07, 0.08 | 0.01 | | -0.02, 0.04 |
| Comorbidities |  |  |  | |  |
| No | — | — | — | | — |
| Yes | -0.09 | -0.14, -0.04 | -0.10 | | -0.12, -0.07 |
| Diet Diversity Score^3^ | 0.05 | 0.03, 0.07 | 0.05 | | 0.05, 0.06 |

CI = Confidence Interval; ^3^Breastfeed within one hour of birth; ^4^Refers to foods or liquids other than breast milk that were given to infants within the first 3 days of their birth; ^5^Diet diversity score of children aged 6 - 23 months

**Supplementary Table 6:** Multiple linear regression on HAZ for all children aged 6 to 59m

|  | NFHS-3 | | NFHS-4 | |
| --- | --- | --- | --- | --- |
| Characteristic | Regression Coefficient | 95% CI | Regression Coefficient | 95% CI |
| Age in Years |  |  |  |  |
| <1 Year | — | — | — | — |
| 1-3 Year | -1.0 | -1.0, -0.91 | -0.82 | -0.85, -0.80 |
| 3-5 Year | -0.89 | -1.0, -0.83 | -0.86 | -0.89, -0.83 |
| Residence Type |  |  |  |  |
| Urban | — | — | — | — |
| Rural | 0.06 | 0.01, 0.10 | 0.03 | 0.01, 0.05 |
| Mother's Education |  |  |  |  |
| No Education | — | — | — | — |
| Primary | 0.15 | 0.09, 0.21 | 0.10 | 0.08, 0.13 |
| Secondary | 0.31 | 0.26, 0.36 | 0.30 | 0.28, 0.32 |
| Higher | 0.70 | 0.61, 0.79 | 0.54 | 0.50, 0.58 |
| Wealth Index |  |  |  |  |
| Poorest | — | — | — | — |
| Poorer | 0.19 | 0.13, 0.26 | 0.20 | 0.18, 0.23 |
| Middle | 0.35 | 0.28, 0.42 | 0.40 | 0.37, 0.43 |
| Richer | 0.57 | 0.50, 0.64 | 0.58 | 0.55, 0.61 |
| Richest | 0.95 | 0.86, 1.0 | 0.79 | 0.75, 0.83 |
| Source of drinking water |  |  |  |  |
| Not Improved | — | — | — | — |
| Improved | -0.15 | -0.20, -0.09 | -0.15 | -0.18, -0.12 |
| Sex of child |  |  |  |  |
| Male | — | — | — | — |
| Female | 0.07 | 0.03, 0.11 | 0.12 | 0.10, 0.14 |
| Breastfed within1h^1^ |  |  |  |  |
| Yes | — | — | — | — |
| No | -0.03 | -0.07, 0.02 | -0.07 | -0.09, -0.05 |
| Complementary food in 3 days^2^ |  |  |  |  |
| Given Nothing | — | — | — | — |
| Given something | -0.09 | -0.13, -0.05 | -0.04 | -0.06, -0.02 |
| Sex of head of household |  |  |  |  |
| Male | — | — | — | — |
| Female | 0.03 | -0.03, 0.09 | -0.03 | -0.05, 0.00 |
| Comorbidities |  |  |  |  |
| No | — | — | — | — |
| Yes | 0 | -0.05, 0.04 | -0.04 | -0.07, -0.02 |

CI = Confidence Interval; ^1^Breastfeed within one hour of birth; ^2^Refers to foods or liquids other than breast milk that were given to infants within the first 3 days of their birth.

## **Supplementary Table 7:** Multiple linear regression on WAZ for all children aged 6m to 59 months

|  | NFHS-3 | | NFHS-4 | |
| --- | --- | --- | --- | --- |
| Characteristic | Regression Coefficient | 95% CI | Regression Coefficient | 95% CI |
| Age in Years |  |  |  |  |
| <1 Year | — | — | — | — |
| 1-3 Year | -0.30 | -0.34, -0.26 | -0.26 | -0.28, -0.24 |
| 3-5 Year | -0.38 | -0.42, -0.33 | -0.35 | -0.37, -0.33 |
| Residence Type |  |  |  |  |
| Urban | — | — | — | — |
| Rural | 0.03 | 0.00, 0.07 | 0.06 | 0.04, 0.08 |
| Mother's Education |  |  |  |  |
| No Education | — | — | — | — |
| Primary | 0.15 | 0.10, 0.19 | 0.11 | 0.09, 0.13 |
| Secondary | 0.28 | 0.24, 0.32 | 0.25 | 0.23, 0.26 |
| Higher | 0.60 | 0.53, 0.67 | 0.47 | 0.45, 0.50 |
| Wealth Index |  |  |  |  |
| Poorest | — | — | — | — |
| Poorer | 0.24 | 0.19, 0.29 | 0.24 | 0.22, 0.26 |
| Middle | 0.44 | 0.39, 0.49 | 0.43 | 0.41, 0.45 |
| Richer | 0.62 | 0.57, 0.68 | 0.59 | 0.57, 0.61 |
| Richest | 0.93 | 0.86, 1.0 | 0.77 | 0.74, 0.80 |
| Source of drinking water |  |  |  |  |
| Not Improved | — | — | — | — |
| Improved | -0.08 | -0.12, -0.04 | -0.13 | -0.15, -0.11 |
| Sex of child |  |  |  |  |
| Male | — | — | — | — |
| Female | 0.01 | -0.02, 0.04 | 0.06 | 0.05, 0.07 |
| Breastfed within1hr^1^ |  |  |  |  |
| Yes | — | — | — | — |
| No | -0.06 | -0.09, -0.02 | -0.09 | -0.10, -0.07 |
| Complementary food in 3 days^2^ |  |  |  |  |
| Given Nothing | — | — | — | — |
| Given something | -0.04 | -0.07, -0.01 | 0.02 | 0.01, 0.04 |
| Sex of head of household |  |  |  |  |
| Male | — | — | — | — |
| Female | 0.02 | -0.03, 0.07 | 0.02 | 0.00, 0.04 |
| Comorbidities |  |  |  |  |
| No | — | — | — | — |
| Yes | -0.10 | -0.13, -0.06 | -0.07 | -0.09, -0.06 |

CI = Confidence Interval; ^1^Breastfeed within one hour of birth; ^2^Refers to foods or liquids other than breast milk that were given to infants within the first 3 days of their birth.

## **Supplementary Table 8**: Per-consumption unit (adult female equivalent) intake of milk& milk products in triangulated data of NFHS-3 and NFHS-4

|  | **NFHS-3** | **NFHS-4** |
| --- | --- | --- |
| Milk and milk products (g/day) | Mean ± SD = 143.62 ±163.90  Median= 88.96  (Q1-Q3: 25.97 - 203.87) | Mean ± SD 155.17 ± 168.65  Median= 101.66  (Q1-Q3: 32.47 - 220.84) |

**Supplementary Table 9:** Path analysis coefficients of HAZ

| **Dependent Variable** | **Independent Variable** | **Unstandardized path coefficient (95% CI)** | |
| --- | --- | --- | --- |
|  |  | **NFHS 3 (2004-2005)** | **NFHS 4 (2014-2015)** |
| Residence Type | Wealth Index | 0.181 (0.177, 0.185) | 0.144 (0.142, 0.145) |
| Source of drinking water | Residence Type | 0.139 (0.127, 0.148) | 0.062 (0.059, 0.065) |
| Milk & Milk Products | Wealth Index | 3.338 (3.179, 3.494) | 3.444 (3.375, 3.499) |
| Breastfed within1hr^1^ | Mother's Education | 0.064 (0.059, 0.068) | 0.026 (0.023, 0.029) |
| Complementary food in 3 days^2^ | Mother's Education | -0.068 (-0.074, -0.062) | -0.004 (-0.006, -0.002) |
| HAZ | Source of drinking water | -0.154 (-0.221, -0.109) | -0.126 (-0.153, -0.097) |
| HAZ | Wealth Index | 0.145 (0.129, 0.16) | 0.102 (0.093, 0.11) |
| HAZ | Mother's Height | 0.05 (0.046, 0.053) | 0.052 (0.051, 0.054) |
| HAZ | Milk & Milk Products | 0.001 (0, 0.002) | 0.002 (0.002, 0.003) |
| HAZ | Mother's BMI | 0.036 (0.031, 0.043) | 0.031 (0.028, 0.032) |
| HAZ | Mother's Education | 0.179 (0.149, 0.201) | 0.16 (0.15, 0.171) |
| HAZ | Sex of child | 0.096 (0.062, 0.139) | 0.132 (0.115, 0.15) |
| HAZ | Comorbidities | 0.053 (0.008, 0.096) | 0.006 (-0.019, 0.023) |
| HAZ | Complementary food in 3 days | -0.084 (-0.126, -0.037) | -0.033 (-0.057, -0.008) |
| HAZ | Breastfed within1hr | 0.024 (-0.017, 0.081) | 0.075 (0.057, 0.092) |

^1^Breastfeed within one hour of birth; ^2^Refers to foods or liquids other than breast milk that were given to infants within the first 3 days of their birth.

**Supplementary Table 10:** Path analysis coefficients of WAZ

| **Dependent Variable** | **Independent Variable** | **Unstandardized path coefficient (95% CI)** | |
| --- | --- | --- | --- |
|  |  | **NFHS-3 (2004-2005)** | **NFHS-4 (2014-2015)** |
| Residence Type | Wealth Index | 0.181 (0.178, 0.184) | 0.144 (0.143, 0.146) |
| Source of drinking water | Residence Type | 0.139 (0.129, 0.147) | 0.062 (0.059, 0.066) |
| Milk & Milk Products | Wealth Index | 3.379 (3.234, 3.512) | 3.348 (3.286, 3.418) |
| Breastfed within1hr^1^ | Mother's Education | 0.064 (0.058, 0.069) | 0.026 (0.024, 0.029) |
| Complementary food in 3 days^2^ | Mother's Education | -0.068 (-0.074, -0.063) | -0.004 (-0.007, -0.002) |
| WAZ | Source of drinking water | -0.072 (-0.104, -0.034) | -0.106 (-0.126, -0.086) |
| WAZ | Wealth Index | 0.14 (0.125, 0.157) | 0.096 (0.09, 0.102) |
| WAZ | Mother's Height | 0.038 (0.035, 0.04) | 0.039 (0.038, 0.04) |
| WAZ | Milk & Milk Products | -0.001 (-0.002, 0) | -0.001 (-0.001, 0) |
| WAZ | Mother's BMI | 0.062 (0.057, 0.067) | 0.056 (0.054, 0.057) |
| WAZ | Mother's Education | 0.143 (0.127, 0.162) | 0.128 (0.121, 0.135) |
| WAZ | Sex of child | 0.015 (-0.01, 0.044) | 0.065 (0.05, 0.075) |
| WAZ | Comorbidities | -0.055 (-0.087, -0.024) | -0.04 (-0.056, -0.03) |
| WAZ | Complementary food in 3 days | -0.03 (-0.063, 0) | 0.023 (0.006, 0.038) |
| WAZ | Breastfed within1hr | 0.059 (0.03, 0.097) | 0.083 (0.068, 0.099) |

^1^Breastfeed within one hour of birth; ^2^Refers to foods or liquids other than breast milk that were given to infants within the first 3 days of their birth.

**Supplementary Table 11:** Path analysis coefficients of Underweight (WAZ < -2 vs WAZ >=-2)

| **Dependent Variable** | **Independent Variable** | **Unstandardized path coefficient (95% CI)** | |
| --- | --- | --- | --- |
|  |  | **NFHS-3** | **NFHS-4** |
| Residence Type | Wealth Index | 0.184 (0.175, 0.193) | 0.148 (0.145, 0.151) |
| Source of drinking water | Residence Type | 0.12 (0.106, 0.133) | 0.049 (0.044, 0.054) |
| Milk & Milk Products | Wealth Index | 2.651 (2.465, 2.838) | 3.369 (3.297, 3.441) |
| Breastfed within1hr^1^ | Mother's Education | 0.068 (0.051, 0.085) | 0.041 (0.024, 0.058) |
| Complementary food in 3 days^2^ | Mother's Education | -0.07 (-0.129, -0.011) | -0.023 (-0.026, -0.02) |
| Underweight | Source of drinking water | -0.021 (-0.07, 0.029) | 0.008 (-0.017, 0.032) |
| Underweight | Wealth Index | -0.063 (-0.081, -0.046) | -0.037 (-0.044, -0.029) |
| Underweight | Mother's Height | -0.01 (-0.013, -0.007) | -0.008 (-0.009, -0.006) |
| Underweight | Milk & Milk Products | 0.001 (0, 0.003) | 0 (0, 0.001) |
| Underweight | Mother's BMI | -0.056 (-0.062, -0.05) | -0.046 (-0.048, -0.043) |
| Underweight | Mother's Education | -0.043 (-0.066, -0.019) | -0.027 (-0.036, -0.018) |
| Underweight | Sex of child | -0.059 (-0.096, -0.021) | -0.07 (-0.086, -0.055) |
| Underweight | Comorbidities | 0.114 (0.073, 0.156) | 0.03 (0.012, 0.049) |
| Underweight | Complementary food in 3 days | -0.005 (-0.043, 0.033) | -0.064 (-0.084, -0.045) |
| Underweight | Breastfed within1hr | -0.038 (-0.08,0.004) | -0.017 (-0.032, 0.001) |

^1^Breastfeed within one hour of birth; ^2^Refers to foods or liquids other than breast milk that were given to infants within the first 3 days of their birth.

**Supplementary Table 12:** Path analysis coefficients of Stunting (HAZ < -2 vs HAZ >=-2)

| **Dependent Variable** | **Independent Variable** | **Unstandardized path coefficient (95% CI)** | |
| --- | --- | --- | --- |
|  |  | **NFHS-3** | **NFHS-4** |
| Residence Type | Wealth Index | 0.184 (0.175, 0.192) | 0.148 (0.146, 0.151) |
| Source of drinking water | Residence Type | 0.118 (0.103, 0.133) | 0.049 (0.044, 0.054) |
| Milk & Milk Products | Wealth Index | 2.976 (2.786, 3.166) | 3.451 (3.378, 3.524) |
| Breastfed within1hr^1^ | Mother's Education | 0.068 (0.051, 0.085) | 0.041 (0.024, 0.058) |
| Complementary food in 3 days^2^ | Mother's Education | -0.07 (-0.13, -0.01) | -0.023 (-0.026, -0.02) |
| Stunting | Source of drinking water | 0.11 (0.066, 0.153) | 0.085 (0.063, 0.108) |
| Stunting | Wealth Index | -0.115 (-0.13, -0.099) | -0.081 (-0.088, -0.075) |
| Stunting | Mother's Height | -0.041 (-0.044, -0.038) | -0.042 (-0.043, -0.041) |
| Stunting | Milk & Milk Products | 0 (-0.001, 0.001) | 0 (-0.001, 0) |
| Stunting | Mother's BMI | -0.031 (-0.036, -0.026) | -0.026 (-0.028, -0.024) |
| Stunting | Mother's Education | 0.004 (-0.062, 0.07) | -0.127 (-0.136, -0.119) |
| Stunting | Sex of child | -0.064 (-0.097, -0.032) | -0.081 (-0.095, -0.067) |
| Stunting | Comorbidities | -0.045 (-0.082, -0.007) | -0.009 (-0.026, 0.008) |
| Stunting | Complementary food in 3 days | 2.017 (1.17, 2.863) | 0.03 (0.012, 0.047) |
| Stunting | Breastfed within1hr | -0.049 (-0.084, -0.014) | -0.048 (-0.062, -0.033) |

^1^Breastfeed within one hour of birth; ^2^Refers to foods or liquids other than breast milk that were given to infants within the first 3 days of their birth.

**Supplementary Table 13:** Comparison of milk intake (Statistically matched by NIBAS) with consumption of dairy products for children aged 6-23 months.

|  | **Number of dairy products in last 24 hours (NFHS data)** | | | | |
| --- | --- | --- | --- | --- | --- |
|  | **0** | **1** | **2** | **3** | **P Value** |
| **NFHS 3** | 59 (15, 152) | 106 (34, 218) | 149 (56, 278) | 181 (83, 345) | <0.001 |
| **NFHS 4** | 80 (23, 183) | 120 (43, 241) | 137 (53, 271) | 142 (53, 262) | <0.001 |

Values are daily median (quartile 1, quartile 3) Adult Female Equivalent (AFE) of milk and milk products consumed in gm.
